# ECM-mimetic, NSAIDs loaded thermo-responsive, immunomodulatory hydrogel for rheumatoid arthritis treatment

**DOI:** 10.1186/s12896-024-00856-3

**Published:** 2024-05-09

**Authors:** Dipesh Kumar Shah, Sumanta Ghosh, Namdev More, Mounika Choppadandi, Mukty Sinha, Sarath Babu Srivalliputtur, Ravichandiran Velayutham, Govinda Kapusetti

**Affiliations:** 1 National Institute of Pharmaceutical Education and Research - Kolkata, Chunilal Bhawan, 168, Maniktala Main Road, Kolkata, 700054 India; 2 National Institute of Pharmaceutical Education and Research - Ahmedabad, Opp. Airforce station, Gandhinagar, Gujarat 382355 India; 3 Siemens Healthcare Pvt. Ltd, Hosur, Bangalore, Karnataka 560100 India

**Keywords:** Injectable hydrogel, Immunomodulatory hydrogel, Intra-articular pain management, Rheumatoid arthritis

## Abstract

**Background:**

Rheumatoid arthritis (RA) is a chronic inflammatory autoimmune disease, and it leads to irreversible inflammation in intra-articular joints. Current treatment approaches for RA include non-steroidal anti-inflammatory drugs (NSAIDs), disease-modifying anti-rheumatic drugs (DMARDs), corticosteroids, and biological agents. To overcome the drug-associated toxicity of conventional therapy and transdermal tissue barrier, an injectable NSAID-loaded hydrogel system was developed and explored its efficacy.

**Results:**

The surface morphology and porosity of the hydrogels indicate that they mimic the natural ECM, which is greatly beneficial for tissue healing. Further, NSAIDs, i.e., diclofenac sodium, were loaded into the hydrogel, and the in vitro drug release pattern was found to be burst release for 24 h and subsequently sustainable release of 50% drug up to 10 days. The DPPH assay revealed that the hydrogels have good radical scavenging activity. The biocompatibility study carried out by MTT assay proved good biocompatibility and anti-inflammatory activity of the hydrogels was carried out by gene expression study in RAW 264.7 cells, which indicate the downregulation of several key inflammatory genes such as COX-2, TNF-α & 18s.

**Conclusion:**

In summary, the proposed ECM-mimetic, thermo-sensitive in situ hydrogels may be utilized for intra-articular inflammation modulation and can be beneficial by reducing the frequency of medication and providing optimum lubrication at intra-articular joints.

**Graphical Abstract:**

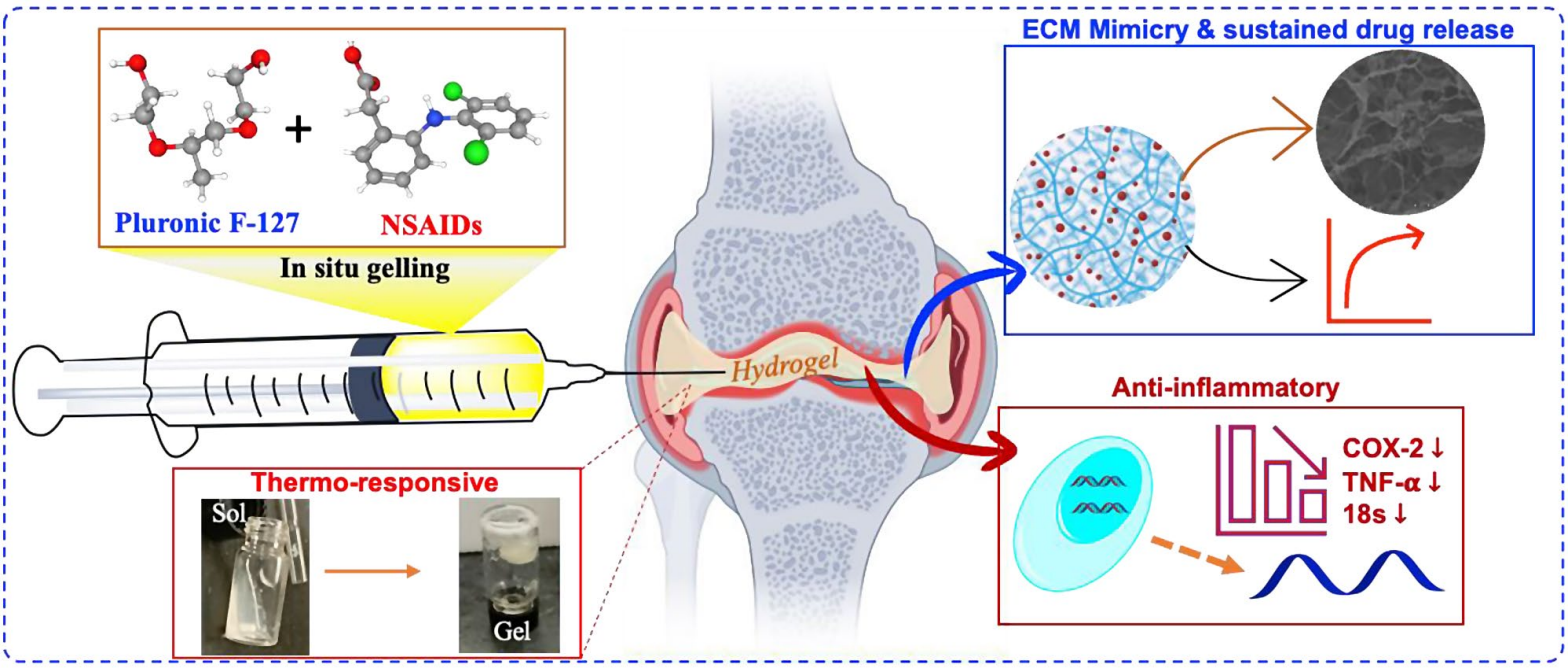

**Supplementary Information:**

The online version contains supplementary material available at 10.1186/s12896-024-00856-3.

## Introduction

Rheumatoid Arthritis (RA) is one of the most common devastating autoimmune diseases, primarily characterized by severe inflammation at the synovial junctions and results in intolerable joint pain and stiffness, leading to severe disability and premature mortality [1, 2]. According to WHO, presently, about 18 million people are suffering from RA all over the world, and among them, 80% of patients experience moderate to severe pain, which leads to rehabilitation [1, 3]. Current therapeutic interventions for RA include oral, parenteral, and site-directed delivery of non-steroidal anti-inflammatory drugs (NSAIDs), disease-modifying anti-rheumatic drugs (DMARDs), and other biological molecules such as stem cells, exosomes, etc [4–6]. Moreover, the current management strategies in the treatment of rheumatoid are focused on “Treat to Target” [7]. However, contemporary treatment options pose major limitations such as symptomatic relief, high drug dose, frequent medication, and impermanent cure leading to joint replacement surgery [7, 8]. NSAIDs, specifically aceclofenac, diclofenac, and ibuprofen, have shown clinically proven efficacy in the management of RA due to their chronic pain relief and potent anti-inflammatory action by inhibiting prostaglandin synthesis [9, 10]. Among them, diclofenac has high potential because of its mild acidic nature with low pK values, which tends to accumulate at the inflamed synovial fluid region [11, 12]. However, like most NSAIDs, it also has a short plasma half-life and extreme potency, which can result in some unwanted effects such as gastric bleeding, impaired kidney functions, etc [12, 13]. Therefore, there is an unmet clinical need to deliver these therapeutic agents in a specific local area and well-controlled manner.

**Fig. 1:**
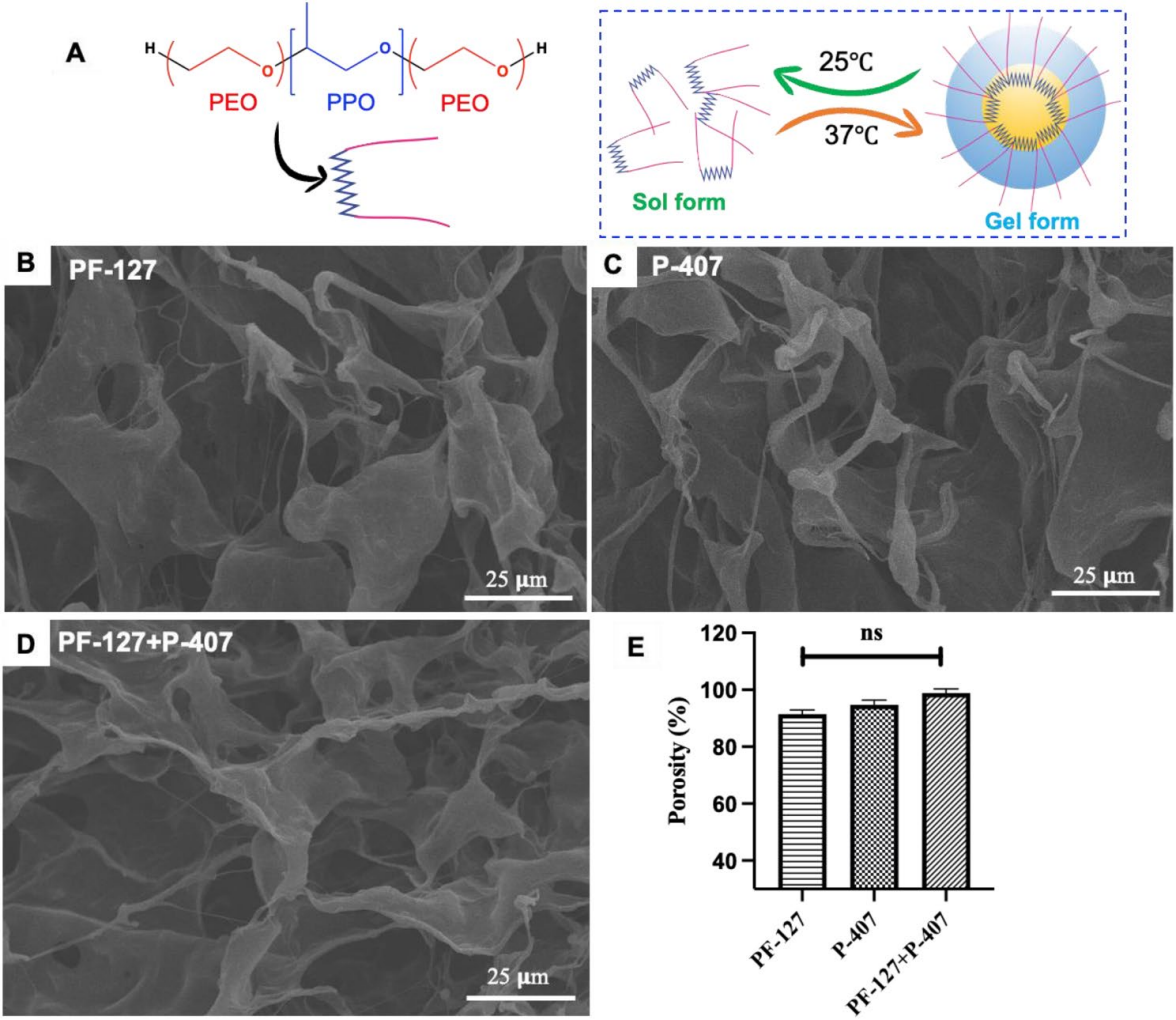
(**A**) Illustration of thermo-responsive gel formation by PF-127/P-407, (**B**-**D**) Representative SEM images of the corresponding hydrogel, (**E**) Quantification of porosity of the fabricated hydrogel

Hydrogel is a hydrophilic macromolecular 3-dimensional cross-linked polymeric network that swells in water or biological fluids [14, 15]. It has the ability to retain a large amount of water due to the presence of the porous microstructure ranging from several nanometers to micrometers [14, 16]. Additionally, it has been proven that the 3D porous network of the hydrogel mimics the extracellular matrix of natural body tissues, which is greatly beneficial for the tissue healing process [17]. Furthermore, *in-situ* hydrogels are highly desirable in RA as it is flowable prior to injection, and upon administration, they get converted into a gel that fits the irregular joint structure [18, 19]. Nevertheless, in recent times stimuli-responsive hydrogel systems have widely explored as a delivery platform, as they can tun the release amount of potent anti-rheumatoid drugs depending on the various stimuli such as light, pH, temperature, Reactive oxygen species (ROS) level, presence of specific enzymes and so on [20, 21]. Likewise, thermo-sensitive hydrogels are a special class of hydrogel that can change its physiochemical properties in response to body temperature [22, 23]. Materials such as poly N-isopropyl acrylamide, poly- (acrylic acid) (PNIPAm), chitosan, hyaluronic acid, poly (lactide-co-glycolide)–block-poly(ethylene glycol)–block-poly(lactide-co-glycolide) PLGA-PEG-PLGA or PEG-PLGA-PEG and poly-(ethylene oxide)–poly (propylene oxide)–poly-(ethylene oxide) (PEO-PPO-PEO) are best known for thermosensitive hydrogels [20, 21]. Among them, Pluronic F-127 (PF-127) and Poloxamer 407 (P-407) are explored widely for various biomedical applications due to their diverse properties [24–26]. Structurally, both the Pluronic F-127 and Poloxamer 407 contain the same repeated units of PEO-PPO-PEO (i.e., A-B-A triblock polymer). However, they vary the molecular weight and the number of chain repetitions depending on the monomer ratio and synthesis condition [27, 28]. To note, the molecular weights of PF-127 and P407 are 12,600 Da and 14,600 Da, respectively [28, 29]. In general, polypropylene oxides show both hydrophilic and lipophilic properties. When the temperature reaches above the critical micelle concentration, it forms hydrogel by agglomeration (ethylene oxide units come to the outer region, and propylene oxide units drive to the inner region), while in a lower temperature, below the critical micelle concentration (i.e., CMC), the polymer chains separated and come to liquid form (Fig. 1A) [25, 28]. The solution of Pluronic F-127 and Poloxamer-407 remains in liquid form at 28 °C and converts to a semi-solid rigid gel at 37 °C [25, 26]. Due to the sol-gel transition, the solution can easily be injected into the targeted site, and it translates to gel in situ at body temperature. Further, this temperature-dependent sol-to-gel transition also gives advantages to releasing the entrapped therapeutic agents in a precisely controlled sustain release manner [22].

Prior studies show that during arthritis, Cyclooxygenase-2 (COX-2), Tumor Necrosis Factor-α (TNF-α), inducible Nitric Oxide Synthase (iNOS), and 18 S ribosomal RNA play key roles in promoting inflammation by inducing the NF-κβ pathway [1, 30]. Furthermore, these inflammatory markers also upregulate several other pro-inflammatory cytokines, such as interleukin-1- 1, 6, and 17, induced vascularization, and increased vascular permeability, which accelerates disease progression [1, 2]. Therefore, the delivery of NSAIDs, such as diclofenac, is predominantly crucial to inhibit these molecular pathways to modulate inflammation at the RA region. Nonetheless, during arthritis, the inflamed tissue produces a large number of free radicals, predominantly Reactive Oxygen Species (ROS), which rapidly interact with the healthy cells present in the arthritis niche, enhance the severity of the inflammation, and initiate the autoimmune disease [31, 32]. Thus, researchers have also delivered various kinds of antioxidant molecules to tackle this issue. For instance, Hyeon et al. [33] developed a Manganese Ferrite/Ceria co-decorated nanoparticles for the scavenging of ROS and synergistically generate oxygen species to repolarize the macrophage for eliciting an anti-inflammatory effect. However, such uncontrollable oxygen generation by the metal nanoparticles in the RA niche resulted in increased oxidative stress in the synovial microenvironment and led to abnormal redox signaling downstream pathways. Therefore, controlled ROS scavenging by targeting the actual inflammatory pathways would be a better approach for minimizing such adverse effects [2, 5].

Here, in this present study, we have developed an ECM mimetic, injectable hydrogel combining thermo-responsive polymers, i.e., Pluronic F-127 (PF-127) and Poloxamer 407 (P-407), and loaded with diclofenac sodium to modulate the chronic inflammatory conditions by inhibiting the expression of several pro-inflammatory genes for the treatment of RA. In addition, we have also explored the controlled free radical scavenging activity of the developed hydrogel to overcome the oxidative stress-dependent inflammatory responses.

## Materials & methodology

### Materials

Pluronic® F-127 (catalog no. P3000MP, MW 12,600 Da, ThermoFisher scientific), commercial poloxomer 407 (BASF Pharma, NJ, Kolliphor® P 407 Geismar, MW 14,600, ≥ 99.0%), 1,1-diphenyl-2-picrylhydrazyl reagent (Sigma Aldrich, ACS reagent, ≥ 99.0%), Lithium bromide (Sigma Aldrich, INDIA), and Butylated hydroxytoluene (ACS reagent, ≥ 99.0%), all the primers were purchased from Sigma-Aldrich, India. Diclofenac sodium (DS) is a generous gift obtained from Cadila Pharmaceuticals, Ahmedabad, India. MTT reagent procured from HiMedia, India. DMEM media (Dulbecco’s Modified Eagle’s medium), Fetal Bovine Serum, and Trypsin EDTA are the products of Gibco® Thermo Fisher Scientific. iScript cDNA synthesis kit purchased from Bio-Rad, Mumbai, India. Methanol, DMSO, potassium chloride, Sodium hydroxide, Sodium dihydrogen phosphate, potassium dihydrogen phosphate, and all other chemicals used in the experiments are of ACS analytical grade (purity > 98%). The purified water from a Milli®-Q Biocel, Millipore® (USA) assembly was used in all the experiments.

### Fabrication of NSAIDs-loaded hydrogel

A range of different concentration (Table S-1) of Pluronic F-127, P-407 were tested for the preparation of optimized in situ thermosensitive hydrogel by using a thermo-gelation process [34]. After that, polymer solutions were prepared in ice-cold water with continuous stirring at 4 °C until a clear solution was obtained and placed in a refrigerator at 4 °C overnight. Later, the solution was kept in a water bath (37℃) where the temperature was increased slowly, and the gelation point was observed with the help of a thermometer. The liquid formulation starts converting from sol to gel at a particular temperature, which is noted as gelation temperature (T_gel_). Similarly, to fabricate the respective NSAIDs-loaded hydrogel, at first 2% (w/v) DS was first dissolved in ice-cold PBS, and next appropriate amount of Pluronic F-127, or P-407 was added into the solution, followed by keeping at 37℃.

### Physiochemical characterizations of hydrogels

#### Surface morphology evaluation

The surface morphology and micro-architecture of the fabricated hydrogel were examined by using Scanning Electron Microscopy (SEM, Hitachi, Japan). Briefly, 100 µl of hydrogel solution was placed on a cover slide and lyophilized. After that, the SEM images were acquired with gold-sputtering [35]. Nevertheless, the porosity of the hydrogel was determined by measuring the pore size of the individual hydrogel by ImageJ software. For the statistical analysis, at least three specimens were used for the same group.

### ATR-FTIR spectroscopy

The drug-polymer compatibility study was carried out by using Attenuated total reflectance-Fourier Transform Infrared spectroscopy (ATR-FTIR) [36]. The functional group of the drug and polymer interaction study was carried out using ATR-FTIR, Bruker Alpha, equipped with Opus software. In frequency range 4000 –400 cm^− 1^ at room temperature. The small quantity of alone diclofenac sodium, polymer, and combination of diclofenac sodium and polymer were placed directly in an ATR-IR sample holder transmittance was taken, and data were analyzed by using software, and the functional group of diclofenac sodium, polymer, and mixture of diclofenac sodium and polymer were analyzed.

### Thermal characterization

The thermal stability of the hydrogel and drug-loaded hydrogel study was examined using Differential scanning calorimetry (DSC 214 Polyma, NETZSCH) [35]. Briefly, 2 mg of hydrogel, with and without drug loaded, was placed in a DSC sample holder. Then, samples were heated at a temperature range of 20–315 °C with a heating rate of 10k/min in a nitrogen atmosphere. The melting temperature in terms of endothermic peak and area under peak (enthalpy) were analyzed using the Polyma software.

### Rheological study of in situ hydrogel

The rheological study of in situ hydrogel was measured with a rheometer (MCR 302, Anton-Paar, Austria) using the 25 mm diameter parallel plate measuring system [35]. The shear viscosity was obtained using a rotational mode of operation of a rheometer at a shear rate of 25 s^− 1^ and temperatures of 28 °C and 37 °C. Dynamic property (Dynamic modulus) was obtained using the oscillation mode of operation. In this mode (amplitude and frequency sweep), 1 mL of hydrogel specimens was subjected to 0.1–100% shear strain, 10 rad/sec angular frequency at a temperature of 37 °C; when the sample is deformed at a particular shear strain, it was taken as a maximum shear which can be applied with angular frequency 100 –0.1 rad/sec, at a temperature 37 °C.

### The sol-gel transition of in situ hydrogel

Different concentration of tri-block polymer was used to check the sol-gel transition by applying a higher temperature in a water bath at 37 °C. Then, it will form a gel, and when the temperature decreases, it converts into a liquid form [35]. It’s also checked by applying temperature in a rheometer at 28-37-28 °C, showing the sol-gel-sol transition. The sol-to-gel transition also depends on the concentration of the polymer; if the concentration of the polymer increases, the gelation temperature of the polymer decreases instantaneously.

### Swelling study

The water absorption capacity of the fabricated hydrogel was assessed by immersing the hydrogel with PBS (pH 7.4) at 37 °C [35, 36]. After predetermined time points, hydrogels were taken out from the medium and gently wiped with tissue paper, and the weight was recorded. The swellable of the hydrogel was determined using the following formula-

$${\rm{Swelling}}\,{\rm{ratio}}\,\left({\rm{\% }} \right)\,{\rm{ = }}\,\left({\left({{{\rm{W}}_{{{\rm{f}}^{\rm{ - }}}}}{{\rm{W}}_{\rm{i}}}} \right){\rm{/}}{{\rm{W}}_{\rm{i}}}} \right){\rm{ \times }}\,{\rm{100}}$$

${\rm{W}}_{i\,}^{}\& {{\rm{W}}_f}$ are the initial and final weight of the sample, respectively.

### In vitro biodegradation study

The degradation pattern of the fabricated hydrogel was tested in the PBS (pH 7.4) environment at 37℃ [25]. Briefly, hydrogels are made with 500 µl of polymer solution and weighted. After that, hydrogels are immersed in 10 ml of PBS. After the specific time interval, hydrogels were taken out and dried, and the percentage of degradation was calculated using the following equation-

$${\rm{Degradation}}\,\left({\rm{\% }} \right)\,{\rm{ = }}\,\left({\left({{{\rm{W}}_{{{\rm{f}}^{\rm{ - }}}}}{{\rm{W}}_{\rm{i}}}} \right){\rm{/}}{{\rm{W}}_{\rm{i}}}} \right){\rm{ \times }}\,{\rm{100}}$$

${\rm{W}}_{i\,}^{}\& {{\rm{W}}_f}$ are the initial and final weight of the sample, respectively.

### Drug release study

In vitro drug release studies of diclofenac sodium loaded in situ hydrogel were performed to investigate using the traditional UV detection method [14, 37]. For the drug release study, 3 ml of drug-loaded (9 mg/ml) hydrogel was filled in a dialysis bag in 50 ml of phosphate buffer (pH 7.4) at a temperature of 37 °C with continuous stirring. At predefined time intervals, 1mL of the sample was collected and diluted with phosphate buffer, followed by being subjected to quantitative analysis using a UV-visible spectrophotometer (UV 1800, Shimadzu) at 276 nm wavelength.

### Free radical scavenging assay

Free radical scavenging activity of diclofenac sodium and diclofenac sodium loaded hydrogel formulations (Diclofenac sodium, DS-PF127, and DS + P407) was measured using 1,1-diphenyl-2-picrylhydrazyl (DPPH) assay [38]. At first, a stock solution of DPPH (0.1 mM) in methanol was prepared, and 10–80 µL of the formulation was added to 100 µL of DPPH stock solution. The reaction mixture was incubated in the dark at room temperature for 30 min, and the absorbance was determined at 517 nm in the multimode reader (Varioskan LUX) (Thermo Fisher Scientific) against the control solution. The radical scavenging efficacy was calculated by the following equation:

$${\rm{Scavenging efficacy}}\,\left({\rm{\% }} \right)\,{\rm{ = }}\,\left({\left({{{\rm{A}}_{{\rm{contro}}{{\rm{l}}^{\rm{ - }}}}}{{\rm{A}}_{{\rm{control}}}}} \right){\rm{/}}{{\rm{A}}_{{\rm{control}}}}} \right){\rm{ \times }}\,{\rm{100}}$$

${{\rm{A}}_{{\rm{control}}}}\,{\rm{and}}\,{{\rm{A}}_{{\rm{control}}}}$ are the absorptions for control and specimens, respectively.

### Cell culture

NIH3T3 mouse fibroblasts, and RAW264.7 cells were obtained from the National Centre for Cell Science, Pune, India. Both, NIH3T3 and RAW264.7 macrophage cells were cultured in Dulbecco’s modified Eagle’s medium (DMEM) with 10% Fetal Bovine Serum (FBS) and 1% penicillin-streptomycin. Cells were incubated at 37 °C in an incubator with 5% CO_2_ supply.

### Biocompatibility study

Biocompatibility of diclofenac sodium loaded in situ hydrogels was measured by using 3-(4,5-dimethylthazol-2yl)-2,5-diphenyltetrazolium bromide (MTT) assay with both NIH 3T3 fibroblasts and macrophages [39]. The cells were grown in a 96-well plate density of 1 × 10^4^ cells/well. After 24 h, cells were treated with different concentrations of diclofenac sodium loaded in situ hydrogel and incubated for another 24 h. After that, MTT solution of 5 mg/mL was added and further incubated for 4 hours at 37 °C. Finely 100 µL of DMSO was added to solubilize formazan crystal. The amount of formazan crystal was determined by measuring absorbance at 575 nm using a multimode plate reader (Varioskan LUX) (Thermo Fisher Scientific). The % cell viability was calculated by following the below formula:

$${\rm{Cell}}\,{\rm{viability}}{\mkern 1mu} \left(\% \right)\,{\mkern 1mu} {\rm{ = }}{\mkern 1mu} \,{{{\rm{OD}}{\mkern 1mu} {\rm{of}}{\mkern 1mu} {\rm{sample}} - {\rm{OD}}{\mkern 1mu} {\rm{of}}{\mkern 1mu} {\rm{blank}}} \over {{\rm{OD}}{\mkern 1mu} {\rm{of}}{\mkern 1mu} {\rm{Control}} - {\rm{OD}}{\mkern 1mu} {\rm{of}}{\mkern 1mu} {\rm{blank}}}} \times 100$$,

where OD = optical density.

### Anti-inflammatory activity evaluation using reverse transcription-polymerase chain reaction (RT-PCR) assay

The RAW264.7 cells were seeded in 6 well plates with a cell density of 1 × 10^4^ cells per well and incubated for 24 h in an incubator. After 24 h, cells were treated with 1 µg/mL concentration of lipopolysaccharides (LPS) in DMEM with 10% FBS then cells were incubated for 24 h. After that, cells were treated with different concentrations of formulation and incubated for 24 h. After 24 h, cells were taken, and RNA was isolated by using the TRIzol method, and cytokinin expression was determined by using RT-PCR [40, 41].

Briefly, Total cellular RNA was isolated from control-treated groups of cells using the TRIzol reagent. Changes in the concentration of mRNA for TNFα, COX-2, iNOS, and 18s were analyzed by RT-PCR. Briefly, the total RNA (0.5 µg) was converted to single-stranded cDNA using iScript cDNA Kits (Bio-Rad). The targeted genes are designed by the program and the target cDNA using the following primers in Table 1. The amplification cycles were 95 °C for 90s initial denaturation, 40 cycles for denaturation at 95 °C for 30 s, annealing at 55 °C and extension at 72 °C for 60 s. After amplification, the amplified product was determined by 2% agarose gel electrophoresis for 1 h at 80 V. Gels were then analyzed by Gel Documentation systems (Bio-Rad).

**Table 1:** Sequences of primers used for RT-PCR study

Primers	Forward (F) and reverse (*R*) sequences	Amplicon size (bp)
**TNF-α**	F: GGCAGGTCTACTTTGGAGTCATTGC R: ACATTCGAGGCTCCAGTGAATTCGG	275
**COX-2**	F: TCTGGAACATTGTGAACAACATC R: AAGCTCCTTATTTCCCTTCACA	198
**18s**	F: CCTTTAACGAGGATCCATTGGA R: CGAGCTTTTTAACTGCAGCAACT	133

### Statistical analysis

All the data is expressed as Mean ± Standard deviation. All the experiments were done in triplicate, and one-way analysis of variance (ANOVA) was used to verify the statistical difference between the groups. Graphs are generated through GraphPad Prism Version 8.5 and Microsoft Excel software. ns, *, and *** represents non-significant, P-value < 0.05 and *P* < 0.001 respectively, which is considered as statistical significance.

## Results and discussion

### Surface morphology & porosity

Fig. 1A depicted the formation of thermos-responsive hydrogel by the PF-127 and P-407 at various temperatures. Typically, above the CMC, the PPO units of the PF-127/P-407 exhibit a sol-gel transition and form the hydrogel by agglomerating hydrophobic units through the hydrophilic-hydrophobic interaction. Further, Fig. 1B-D depicts the SEM images of interconnected porous microstructures of the PF-127, P-407, and combined PF-127 + P-407 (1:1) hydrogels, respectively. Prior evidence indicated that these types of porous interconnect networks mimic the natural extracellular matrix (ECM), which is crucial for cell adhesion and attachment [17]. Additionally, all the hydrogels exhibit more than 90% porosity, which provides a nutrient exchange medium between the cells and improves cell-cell communication (Fig. 1E) [17].

### Optimization of gelation temperature

Usually, the gelation temperature of the thermos-responsive materials principally depends on their molecular chain length and the spatiotemporal group’s composition [35]. As in the case of PF-127 and P-407 polymers, both have the same side groups; however, they are different in their molecular chain length structure [29]. Therefore, to obtain the optimal gelation temperature along with the suitable injectable stable gel composition, we have performed the gelation test with various compositions noted in supplementary Table S-1. Nonetheless, from the observation, we can confirm that the PF-127, P-407, and combined PF-127 + P-407 forms optimized gel formation at 18 wt%, 19 wt%, and 9:10 wt%, respectively. Thus, this evidence confirmed that in the case of poloxamer-based hydrogel, the molecular weights don’t significantly impact the gelation temperature [42, 43]. Furthermore, all these compositions exhibit a smooth sol-to-gel transformation at 28℃ to 37℃, which is crucial for the internal in-situ application.

### ATR-FTIR spectroscopy of fabricated hydrogel

**Fig. 2:**
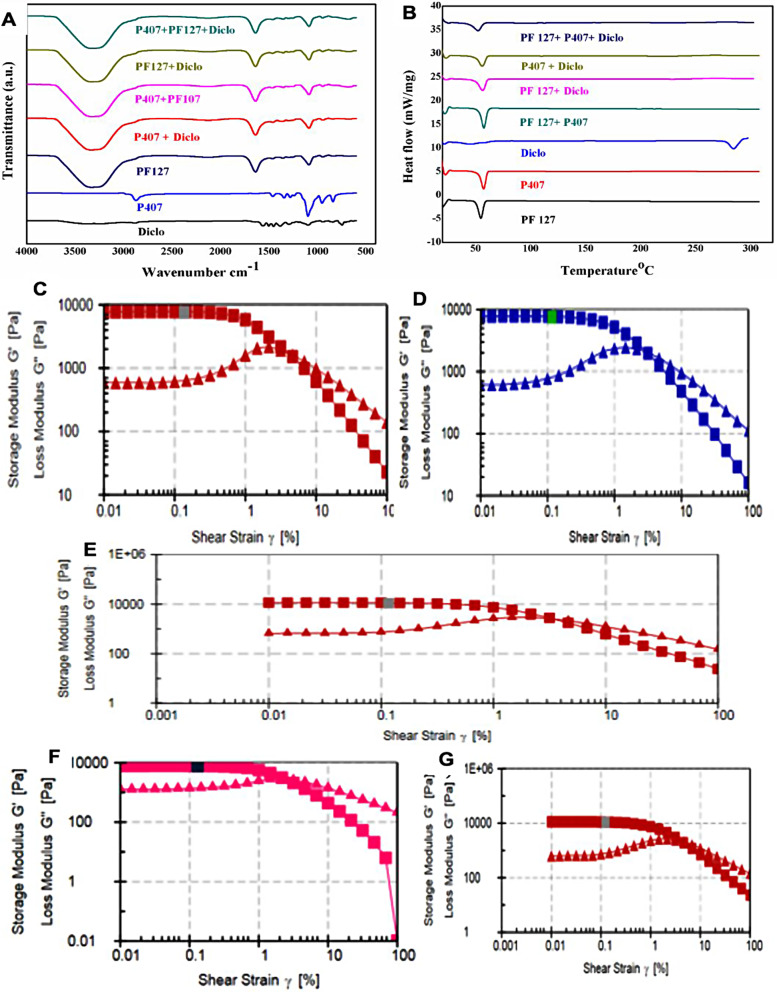
(**A**) FTIR spectra and (**B**) DSC thermograph of diclofenac sodium, P-407, PF-127 alone and in combination form. Rheology studies of hydrogel, (**C**) P-407, (**D**) PF-127, (**E**) P-407 + PF-127, (**F**) Diclofenac sodium loaded P407, and (**G**) DS-loaded PF-127, respectively. These data showed the gel formation and mechanical stability of the fabricated hydrogels

ATR-FTIR was employed to elucidate the chemical interaction between the drug and the polymer within the hydrogel. Fig. 2A represents the FTIR spectra of respective compounds. As observed, the spectra of PF-127 + Diclo, P-407 + Diclo, and combined PF-127 + P-407 + Diclo exhibit the peaks at 2880 cm^− 1^ corresponding to -CH_2,_ 3341 cm^− 1^ corresponding to -NH, 1720 cm^− 1^ corresponding to -C = O, and 1530 cm^− 1^ corresponding to -NH groups present in the backbone of the polymer. Furthermore, the broad peak 3330–3340 cm^− 1^ represents the presence of the -OH group, and the absorption bands at 1638 and 1000–1100 cm^− 1^ denote the presence of C = C and C-O stretching in all the hydrogels [23, 44]. Whereas absorption of 1568 cm^− 1^, 1289 cm^− 1^, and 749 cm^− 1^ denotes the presence of C = O, C-N, and phenyl group confirms the diclofenac sodium [45]. Moreover, all the characteristic peaks of diclofenac sodium and polymer were observed intact, and there is no interaction found in the DS-loaded hydrogel at room temperature.

### Thermal stability of the hydrogel

Thermal stability of the hydrogel composition is one of the important factors for the successful fabrication of thermos-responsive hydrogel. We have examined the thermal stability of the fabricated CS-loaded hydrogel using DSC. Fig. 2B depicts the DSC thermograph of respective compounds and hydrogels. As observed, both the P-407 and PF-127 demonstrated narrow endothermic peaks at ∼ 51.3 to 53.2 °C, respectively, which confirm the thermal stability of the polymer is independent of molecular weight [23, 26]. However, in the case of a drug-loaded hydrogel such as PF-127 + Diclo, P-407 + Diclo, and PF-127 + P-407 + Diclo, the melting temperatures were seen at a higher temperature range of about 55–58.5 °C. This is because, at this temperature range, diclofenac sodium was completely dissolved in the polymer. Thus, the formulation only showed the melting peak of the polymer with the increased enthalpy. Moreover, DSC results confirmed that in all the DS-loaded PF-127, P-407, and PF-127 + P-407 hydrogel, the drug is thermally stable and compatible with the polymer matrix.

### Viscoelastic properties of fabricated hydrogel

The mechanical stability and in situ, gel-forming ability of the fabricated hydrogels were examined by determining the dynamic modulus of the hydrogels using rheology (Fig. S-2). We have performed the amplitude sweep study to evaluate the maximum deformation of the hydrogel with the intersection of storage and loss modulus curves. As depicted in Fig. 2C-E, the deformation of the hydrogels of P-407, PF-127, and combined PF-127 + P-407 has shown shear strain at 0.120, 0.110, and 0.110%, respectively. Nonetheless, when DS was loaded into the hydrogels, the deformation of P-407 and PF-127 was found to be ∼ 0.112 and 0.110%, respectively (Fig. 2F, G). These results indicated that both the pristine P-407, PF-127, and combined PF-127 + P-407 hydrogels and the DS-loaded hydrogels demonstrated similar deformation trends and stability up to ∼ 0.1% strain level.

Furthermore, we have also studied the frequency sweep study to elucidate the stability of the hydrogels with time. As presented in supplementary Fig. S-3, the storage modulus and loss modulus of the PF-127 at higher frequency were constant, but when the frequency was decreased, the storage modulus was decreased, loss modulus was increased, and at a lower frequency, the storage modulus and loss modulus were about to cross. Additionally, P407 hydrogel also exhibits the same pattern, but both moduli tunned parallelly; however, the combination of PF-127 + P407 hydrogel demonstrated stability at a lower frequency, but as the frequency increased, it deformed rapidly. Taken together, rheological analysis reveals that the stability of P407 was more than PF-127, and the combined P407 + PF-127 hydrogels were not physically stable for a longer duration of time compared to the individual hydrogel. This may be due to the fact the addition of different sizes of polymer chains into a specific hydrogel results in more steric hindrances and resistance to the gel formation [14, 16]. Therefore, it is important to mention here that the combined P407 + PF-127 hydrogels don’t exhibit good mechanical stability; thus, for the in vitro experiments, we have only explored the activity of individual drug-loaded P407 and PF-127 hydrogels.

### Viscosity of in situ hydrogel

**Fig. 3:**
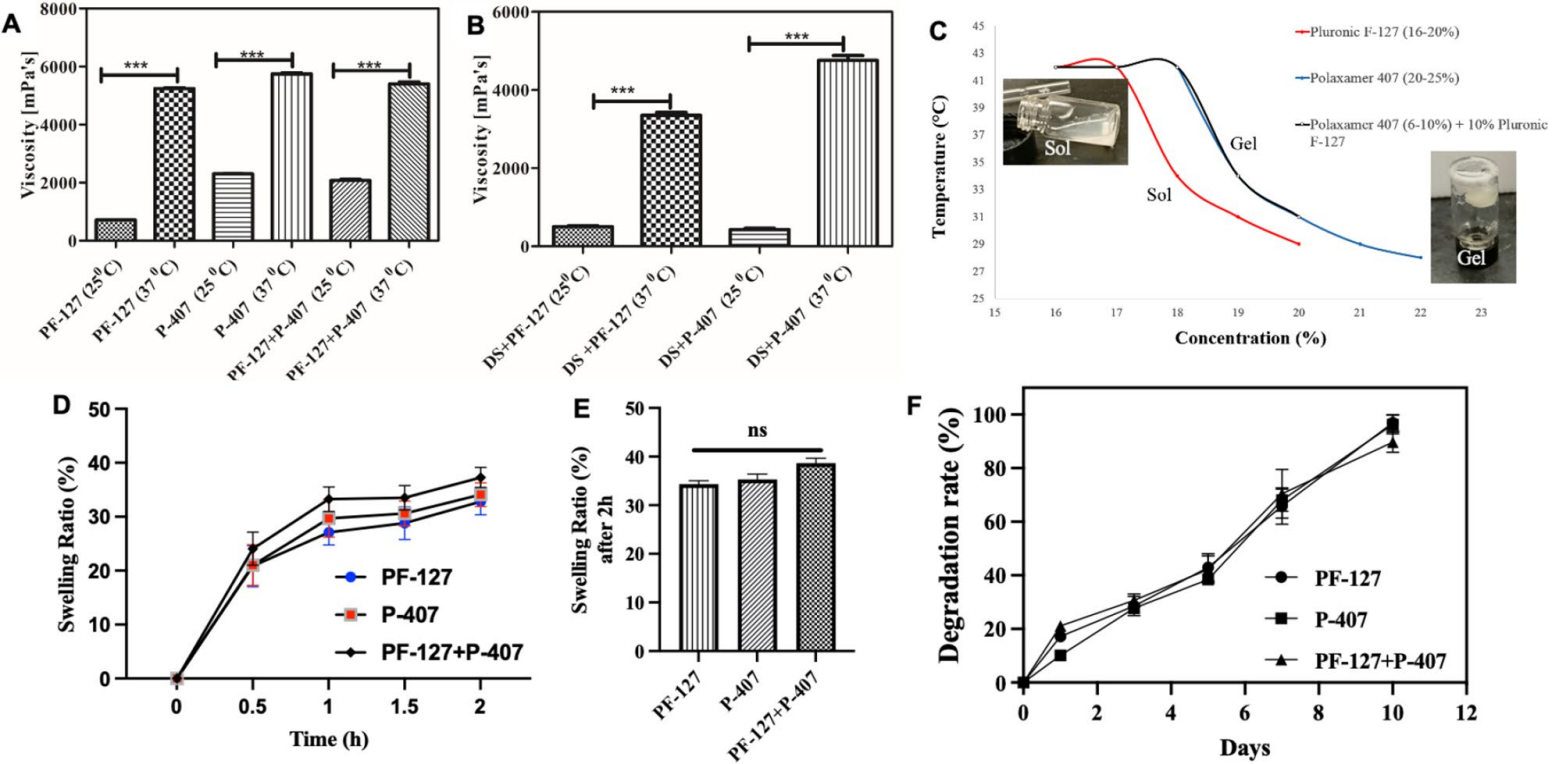
Viscosity of in situ hydrogels. (**A**) Pure in situ hydrogel at 25 and 37 °C, (**B**) Diclofenac sodium (DS) loaded in situ hydrogel at 25 and 37 °C. (**C**) Water swellability of the fabricated hydrogel, (**D**) Statistical comparison of water swellability of the fabricated hydrogel after all the hydrogels attend to their maximum swellability at two h. (**E**) *In-vitro* degradation rate of the fabricated hydrogels showing all the fabricated hydrogels are completely biodegraded within 10 days

Typically, for the shear-thickening polymeric material, the shear viscosity of the hydrogel is directly proportional to temperature [42, 46]. The increase in viscosity was observed when the temperature was increased from 28 °C to 37 °C. The drastic change in viscosity confirmed that the prepared formulation is thermosensitive. It was found that PF-127 showed slightly lower viscosity when compared to P-407 (Fig. 3A & B). In contrast, both DS-loaded hydrogels show slightly decreased viscosity as compared to the pristine gel without the drug. This decreased viscosity may be due to the internal entanglement of drug molecules with polymer chains in a hydrogel, which hindered the sol-gel transition [14].

### The sol-gel transition of in situ hydrogel

Sol-gel transformation is the fundamental characteristic of any thermo-responsive hydrogel. Typically, sol-gel transition depends on the concentration of polymer in the hydrogel; as the concentration of the polymer increases, the gelation occurs at a lower temperature and vice-versa due to the higher interactions of polymer chains. As presented in Fig. 3C, PF-127 demonstrated gelation at 42 °C when the concentration of the solution was 16%, while the 20% solution show shows the gelation at 29 °C. A similar trend was observed in P-407 (22%) and the combination of P-407 + PF-127 (9: 10) at 28–29 °C, respectively. Note that for the in-situ hydrogel fabrication, the smooth sol-gel transition should have occurred between the room temperature and the body temperature. For instance, Dutta et al. [47] reported an in situ forming hydrogel based on PLGA–PEG–PLGA copolymer, which can undergo temperature-induced sol–gel transitions at 25–37 ℃ for the controlled delivery of therapeutic agents. Similarly, herein the fabricated hydrogels exhibit the sol-gel transition between 29 and 37 ℃, confirming its ability to be an in-situ hydrogel suitable for RA application [15, 19].

### Water swellabilty

Due to the presence of porous interconnected network microstructure, hydrogels can adsorb a huge amount of body fluid in their inner matrix. Furthermore, it was also reported that the high swellability also favors the nutrient exchange to the cells embedded in the hydrogel niche. Fig. 3D & E represents the water swellability of the fabricated hydrogel. As observed, all the hydrogel exhibits around 40% swellabilty compared to their initial states within 2 h of incubation. However, after 1.5 h, all the hydrogels attend to their maximum swellability. In addition, Fig. 3E reveals that irrespective of the molecular weights of PF-127 and P-407 and combination (i.e., PF-127 + P-407), hydrogels show the same water swelling capability, which is around 38–42%, favoring the in-situ application and drug release.

### In vitro biodegradation

The biodegradation pattern of the fabricated hydrogels was studied by immersing them in PBS. Fig. 3F represents the degradation patterns of the hydrogel over time. As observed, all the hydrogels, including P-407, PF-127, and P-407 + PF-127, fully degraded within 10 days of incubation. Note that within the initial 3 days, the degradation of hydrogels was very minimal ( > ∼ 20%); however, after the 4th day, a consistent increment of degradation was observed in all the hydrogels. This type of delayed types sustain degradation is very useful for the in situ delivery of hydrogels in the bone/joint area and favors the sustained drug release over a longer period [14, 16].

### In vitro drug loading and drug release characterization

**Fig. 4:**
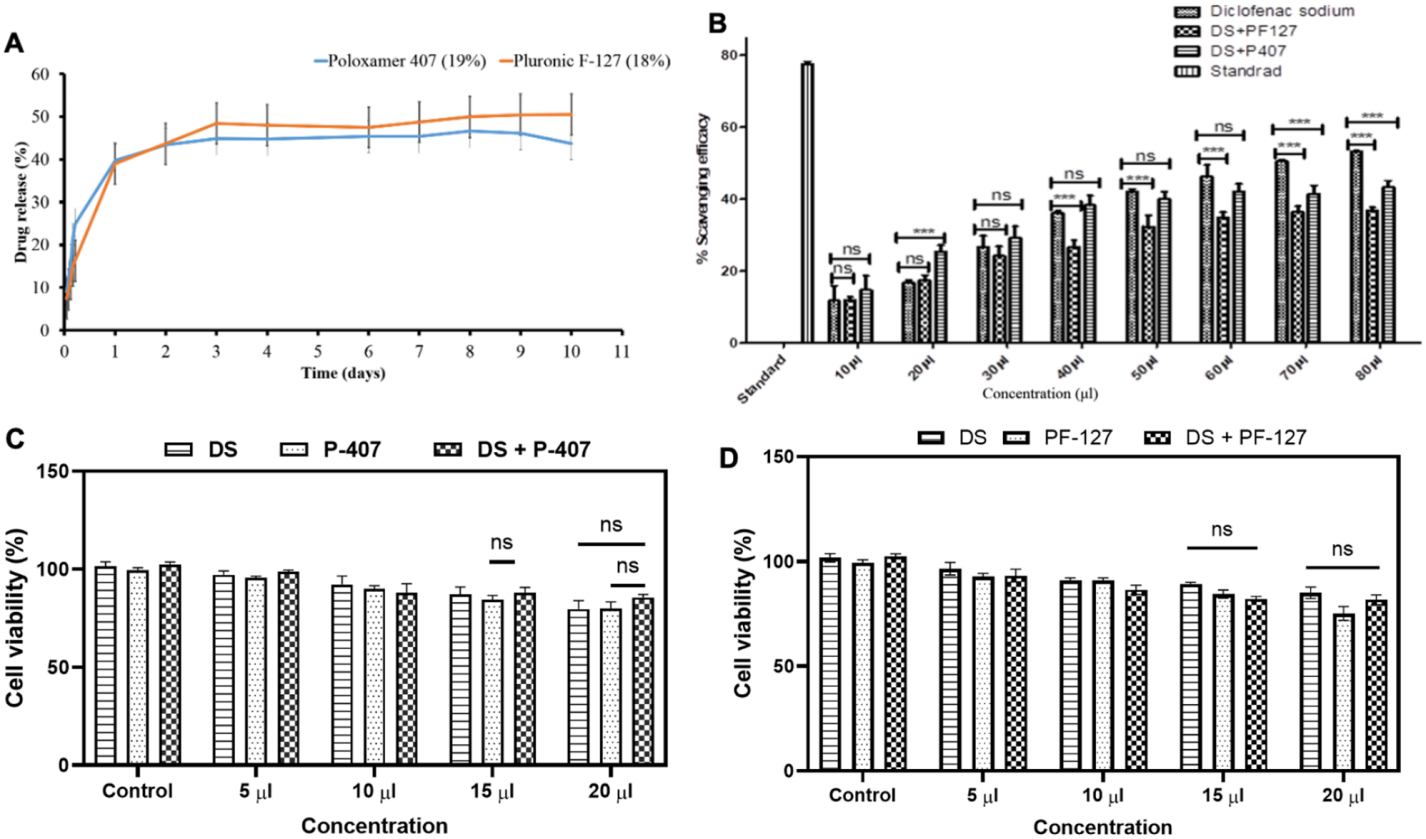
(**A**) Cumulative drug release profile from the thermo-responsive hydrogels, (**B**) Antioxidant activity of the diclofenac loaded thermo-responsive hydrogels, (**C** & **D**) Effect of different concentrations of diclofenac sodium and diclofenac sodium loaded P407 and PF-127 hydrogel on NIH 3T3 cell viability after 24 h incubation

Drug loading was performed by the direct method by incorporation of diclofenac sodium in the prepared hydrogel. The drug release profile of DS was determined in vitro by using phosphate buffer pH-6.8 and 37 °C temperature. When the drug migrated from the polymeric hydrogel in the presence of phosphate buffer, the release was determined by the withdrawal of samples at different intervals. As observed, the DS released from the hydrogels in two phases initially showed burst release within 24 h, and after that, it was sustained over 10 day period (Fig. 4A). Interestingly, this type of initial burst release and successive sustained release pattern is extremely crucial for RA treatment as the initial burst release confirms the higher bioavailability of the drug molecules at the local RA microenvironment to exhibits the potent anti-inflammatory action [48]. Followed by sustained release, maintains the proper drug amounts by preventing wash-out problems through the body fluids [49]. Taken together, our DS-loaded PF-127 + P-407 hydrogels exhibit suitable release patterns for the sustained drug delivery strategy.

### Antioxidant property of fabricated hydrogel

A plethora of prior studies reported that during RA, the generation of various ROS and free radicals from the damaged tissue significantly worsens the inflammatory condition and accelerates the disease progression [31]. Therefore, the application of bioactive scaffolds with free radical scavenging activity is extremely beneficial under those circumstances. Fig. 4B represents the antioxidant properties of the DS-loaded thermo-responsive hydrogels examined by using a traditional DPPH-based free radical scavenging assay. To note, butylated hydroxytoluene (BHT) and pure DS were taken as internal standard and positive control, respectively, in this experiment. As observed, the scavenging activity of both the DS + PF-127 and DS + P-407 hydrogels is the same till 30 µl concentration, in which it shows around 34.8 and 37.4% scavenging, respectively. Whereas, as the concentrations increase by 40–80 µl, we have observed a higher scavenging efficacy. For instance, 80 µl of both DS + PF-127 and DS + P-407 hydrogels exhibit around 44.9% and 47.3% of free radical scavenging. Moreover, these results confirmed that NSAIDs such as DS-loaded PF-127 or P-407-based hydrogels exhibit excellent antioxidant characteristics [31, 32].

### Biocompatibility assessment

Cellular compatibility is one of the most crucial factors for the in-situ hydrogel. Therefore, we have tested the different concentrations of PF-127, P-407, and combined drug-loaded hydrogel composition to optimize the optimal conditions (Fig. 4C & D). As observed, all the hydrogel are extremely biocompatible (viability > 80%) with NIH 3T3 murine fibroblast cells. Further, when co-incubate with macrophages, pure DS exhibits a toxicity level above 17.44 ± 3.62 µM, whereas, in the case of both the DS + P-407 and DS + PF-127, the toxicity level was found to be 19.43 ± 2.96 µM and 23.41 ± 9.88 µM, respectively (Fig. S-4). These results constituently indicated that when DS is incorporated into the PF-127/P-407 hydrogel matrix, it not only modulates its release profile but also increases its biocompatibility. This is important because most of the traditional delivery systems suffer from improper dose dumping effects, which cause the systemic toxicity of the drug molecules [14, 22]. Nonetheless, it was found that till 15 μl concentration, both the DS-loaded hydrogels exhibited more than 90% cell viability. Moreover, this result indicated that DS-loaded hydrogels are fully biocompatible and suitable for RA treatment.

### Anti-inflammatory activity assessment

**Fig. 5:**
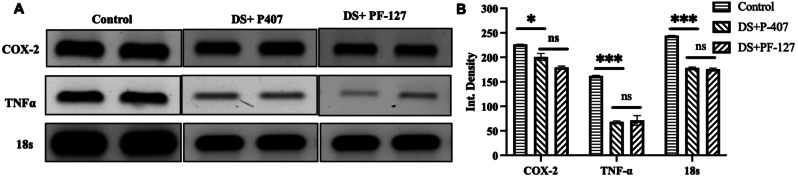
Anti-inflammatory activity of the fabricated DS-loaded hydrogels. (**A**) Representative gel images of the RT-PCR study, (**B**) Quantification of the gene expression level of the respective genes after the hydrogel treatments

The anti-inflammatory characteristics of the fabricated hydrogels were evaluated by RT-PCR examination. To confirm the NSAIDs-mediated anti-inflammatory features of the hydrogel, we have selectively chosen specific makers for inflammation, such as *TNF-α* and *COX-2*, and additionally, a housekeeping gene 18s was also taken into consideration. The PCR results (Fig. 5A & B) revealed that both the DS-loaded PF-407 and P-407 hydrogels were able to significantly decrease the expression of *TNF-α* and *COX-2*, confirming their potent anti-inflammatory activity. Furthermore, in the case of the 18s, similar trends were observed. Together, it has been concluded that DS-loaded PF-407 and P-407 hydrogels exhibit a potent anti-inflammatory activity and, therefore, modulate RA microenvironments for faster healing [33, 50].

## Conclusions

Over the years, dozens of delivery strategies have emerged and applied for the precise delivery of conventional NSAIDs, such as diclofenac, for the treatment of RA. Even so, the majority of them fail to impart significant medical output due to their uncontrollable release profile, dose-dumping effects, unstable nature in the physiological milieu, and many more. Herein, we have proposed a facile fabrication of injectable hydrogels loaded with DS by using thermos-responsive polymers such as PF-127/P-407 to mitigate the inflammatory conditions during the RA. Nonetheless, from the SEM and rheological analysis, we have confirmed that the DS-loaded PF-127 + P-407 hydrogels exhibit a natural ECM-like micro-architecture that supports tissue healing and also has sufficient mechanical stability suitable for the RA treatment. We have also shown that the DS-loaded PF-127 & P-407 hydrogels exhibit a seamless sol-to-gel transformation in the body temperature (28–37℃), which gives advantages like injectability and shape forming capability depending on the irregular synovial cavity [51].

Interestingly, through the amplitude and frequency sweep study, we have revealed that the fabricated hydrogel can also resist the deformation caused by mechanical stress, which is very crucial as, during the RA, circumventing joint pain is necessary. When injected into the joint cavity, this hydrogel swells after absorbing the body fluid and forms a cushion-like scaffold, which minimizes the bone-to-bone contact [51, 52]. Furthermore, through several in vitro studies, we have also verified the biodegradability and biocompatibility of the fabricated hydrogels. Nevertheless, we have also demonstrated that in the physiological milieu, DS-loaded hydrogels exhibit an initial burst release followed by a sustained release profile over 10 days, suitable for the long-term application of RA treatment. We have also explored the antioxidant activity of the fabricated hydrogels, which indicated that DS-loaded PF-127 and P-407 hydrogels also have free radical scavenging activity, preventing tissue damage from ROS. At last, through the RT-PCR, we have confirmed that both of the DS-loaded PF-127 & P-407 hydrogels were able to significantly downregulate the expression of several inflammatory markers such as TNF-α, COX-2, and 18s, confirming its potent anti-inflammatory activity. Moreover, in this study, we have explored the injectable, thermo-responsive, biocompatible, antioxidant, and immune-modulatory properties of NSAIDs-loaded PF-127 P-407 hydrogels for the treatment of RA. We hope that this study will pave the way for developing multifunctional stimuli-responsive biomaterials for circumventing RA as well as other diseases where traditional approaches failed.

### Electronic supplementary material

Below is the link to the electronic supplementary material.

Supplementary Material 1

Supplementary Material 2

## Data Availability

The data used to support the findings of this study are available from the corresponding author upon request.
