# Supplementary material for: ECM-mimetic, NSAIDs loaded thermo-responsive, immunomodulatory hydrogel for rheumatoid arthritis treatment
Source: BMC Biotechnol. 2024 May 9;24:26. doi: 10.1186/s12896-024-00856-3 (PMC11080159; doi:10.1186/s12896-024-00856-3)
Supplement: Supplementary file 1 — Supplementary Material 1 [file 12896_2024_856_MOESM1_ESM.docx]

**Supplementary information**

**ECM-mimetic, NSAIDs Loaded Thermo-responsive, Immunomodulatory Hydrogel for Rheumatoid Arthritis Treatment**

Dipesh Kumar Shah^2, †^, Sumanta Ghosh^2, †^, Namdev More^2^, Mounika Choppadandi^2^, Mukty Sinha^3^, Srivalliputtur Sarath Babu^1^, Ravichandiran Velyutham^1^, Govinda Kapusetti ^1,2,^*

^1^Department of Medical Devices, National Institute of Pharmaceutical Education and Research - Kolkata, Chunilal Bhawan, 168, Maniktala Main Road, Kolkata, INDIA – 700054

^2^Department of Medical Devices, National Institute of Pharmaceutical Education and Research - Ahmedabad, Opp. Airforce station, Gandhinagar, Gujarat, INDIA-382355

^3^Siemens Healthcare Pvt. Ltd, Hosur, Bangalore 560100. Karnataka, India.

^†^ These authors contributed equally to this work

* *Corresponding Author*

Dr. Govinda Kapusetti

Associate Professor

Department of Medical Devices

NIPER-Kolkata, India

E-mail address: [govindphysics@gmail.com](mailto:govindphysics@gmail.com)

**Table S-1.** Different concentrations of PF-127, P-407 and combined PF-127+P-407 hydrogels for the optimization for the gelation temperature

| **Type of polymer** | **Concentration (%, w/w)** | **Observation** | | **T_gel_ (± 1˚C)** |
| --- | --- | --- | --- | --- |
|  |  | **At 28˚C** | **At 37˚C** |  |
| **PF-127** | 16 | liquid | liquid | > 42 |
|  | 17 | liquid | liquid | >42 |
|  | **18** | **liquid** | **gel** | **34** |
|  | 19 | gel | gel | 31 |
|  | 20 | gel | gel | 29 |
| **P407** | 17 | Liquid | Liquid | >42 |
|  | 18 | Liquid | Liquid | >42 |
|  | **19** | **Liquid** | **Gel** | **34** |
|  | 20 | viscous | Gel | 31 |
|  | 21 | Gel | Gel | 29 |
|  | 22 | Gel | Gel | 28 |
| **P407**  **+**  **PF-127** | 1+18 | Gel | Gel | 29 |
|  | 5+18 | Gel | Gel | 26 |
|  | 10+18 | Gel | Gel | 22 |
|  | 15+18 | Gel | Gel | 19 |
|  | 18+18 | Gel | Gel | 18 |
|  | 5+10 | Liquid | Liquid | >42 |
|  | 6+10 | Liquid | Liquid | >42 |
|  | 7+10 | Liquid | Liquid | >42 |
|  | 8+10 | Liquid | Liquid | >42 |
|  | **9+10** | **Liquid** | **Gel** | **34** |
|  | 10+10 | Viscous | Gel | 31 |

**
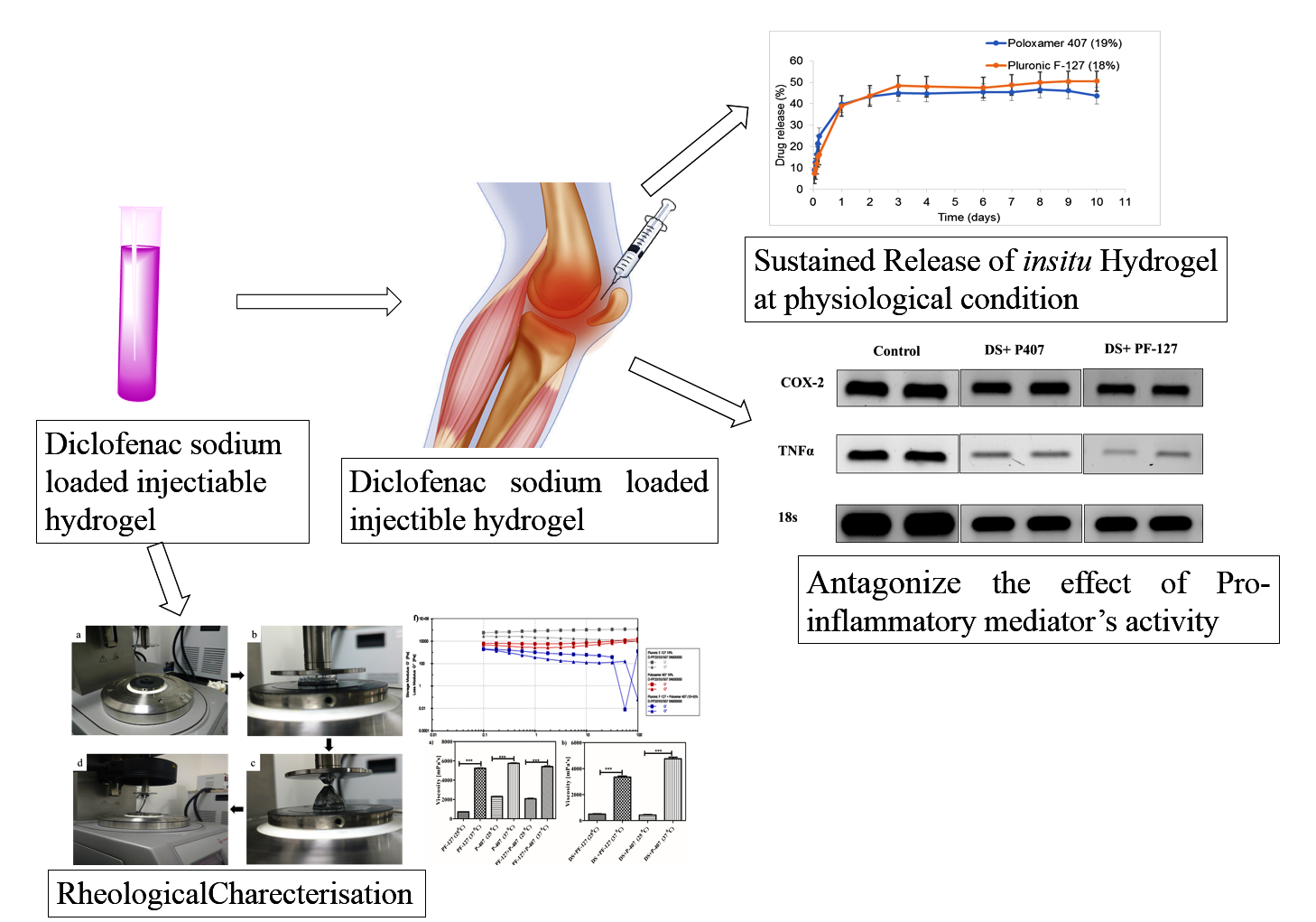
**

**Fig. S-2.** Digital photographs of the viscosity testing of the fabricated hydrogels


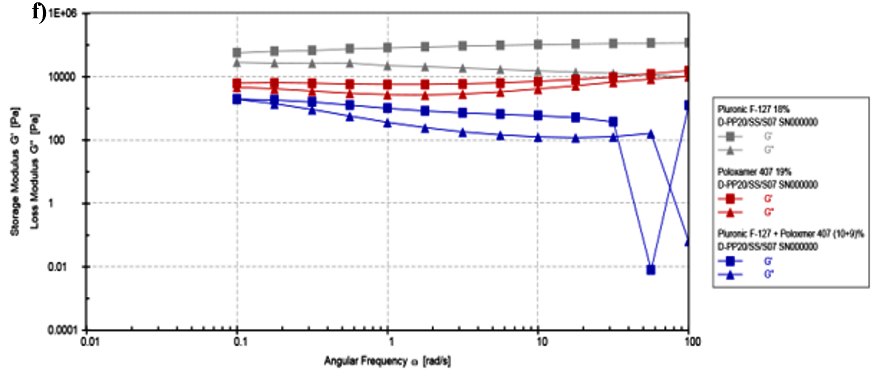


**Fig. S-3.** Frequency sweep testing of the fabricated hydrogels. The results indicated that the storage modulus and loss modulus of the PF-127 at higher frequencies were constant, but when the frequency was decreased, the storage modulus was decreased, loss modulus was increased

**
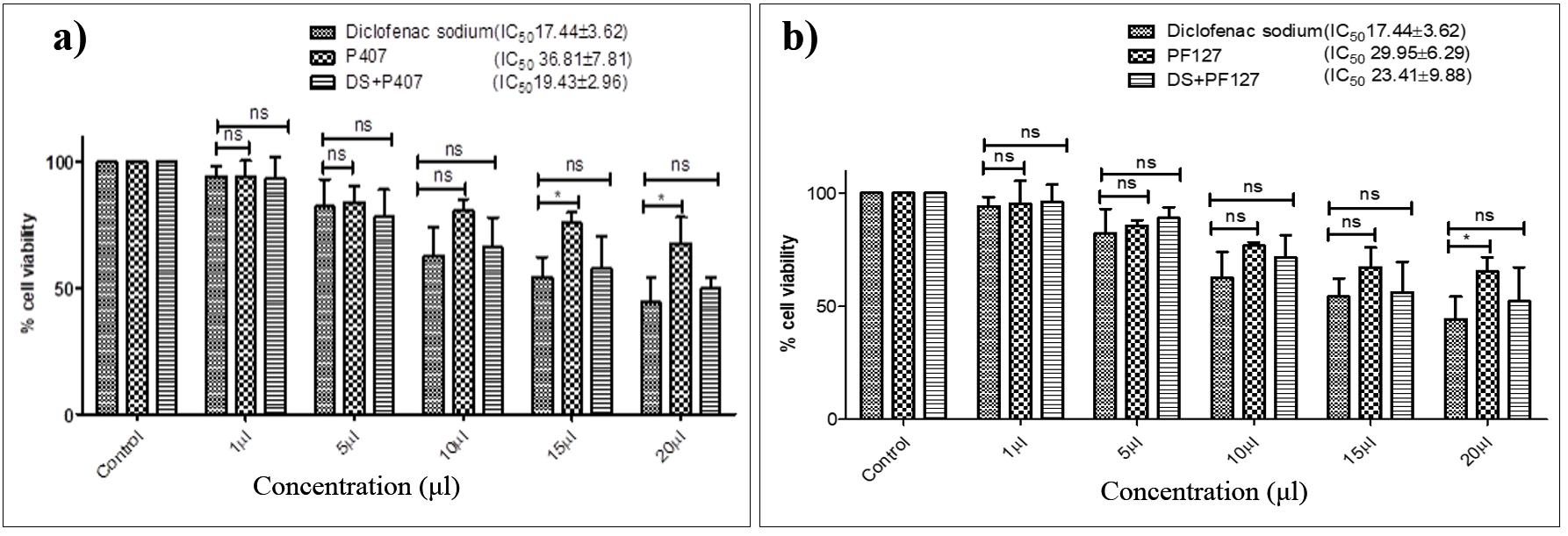
**

**Fig. S-4.** Effect of different concentrations of diclofenac sodium and diclofenac sodium loaded P407 and PF-127 hydrogel on macrophage (RAW264.7) cell viability after 24 hours incubation.


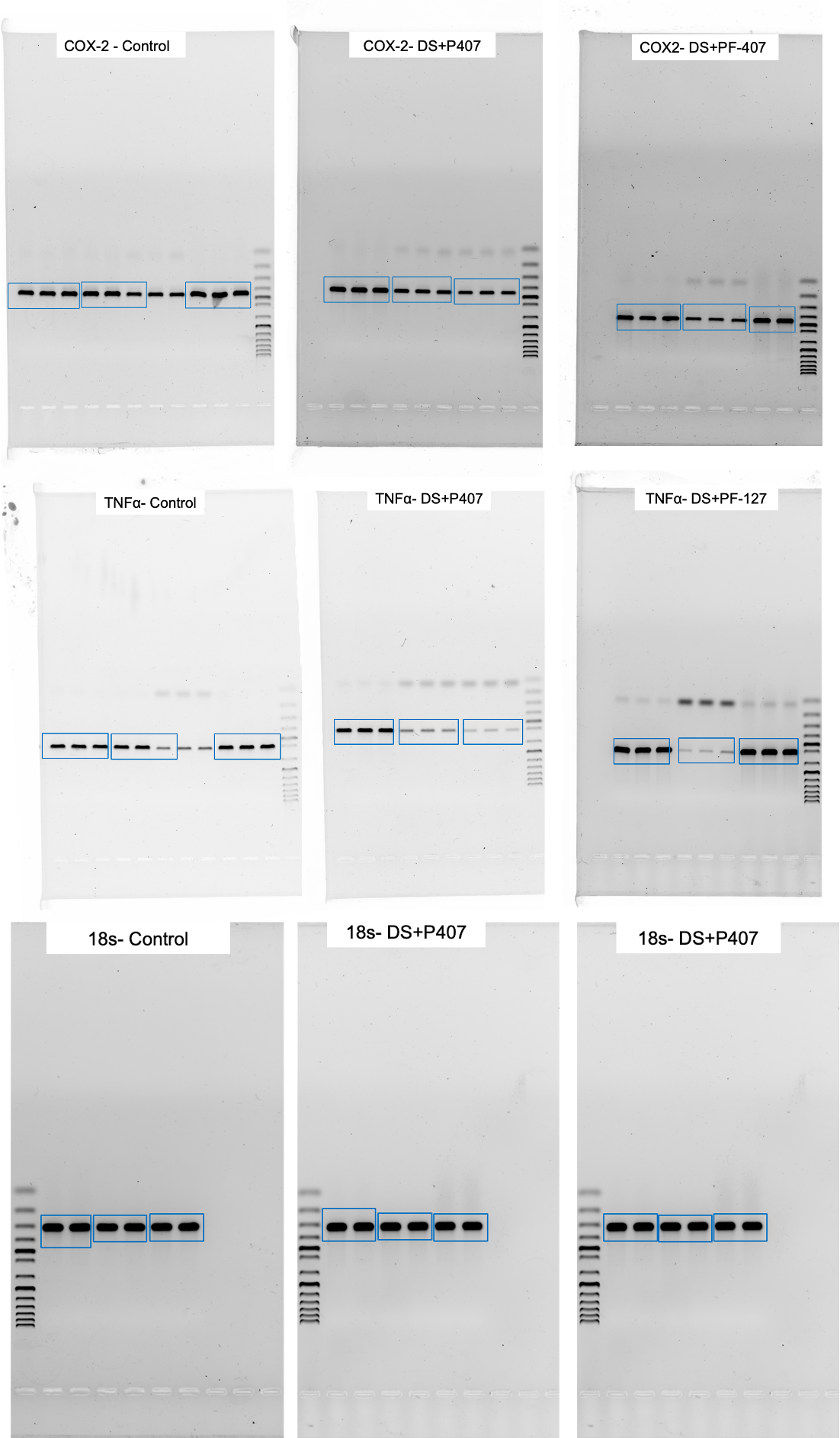


**Fig. S-5.** Full-length RT-PCR gel images of the respective genes.
