# Supplementary figures and images for: ECM-mimetic, NSAIDs loaded thermo-responsive, immunomodulatory hydrogel for rheumatoid arthritis treatment
Source: BMC Biotechnol. 2024 May 9;24:26. doi: 10.1186/s12896-024-00856-3 (PMC11080159; doi:10.1186/s12896-024-00856-3)

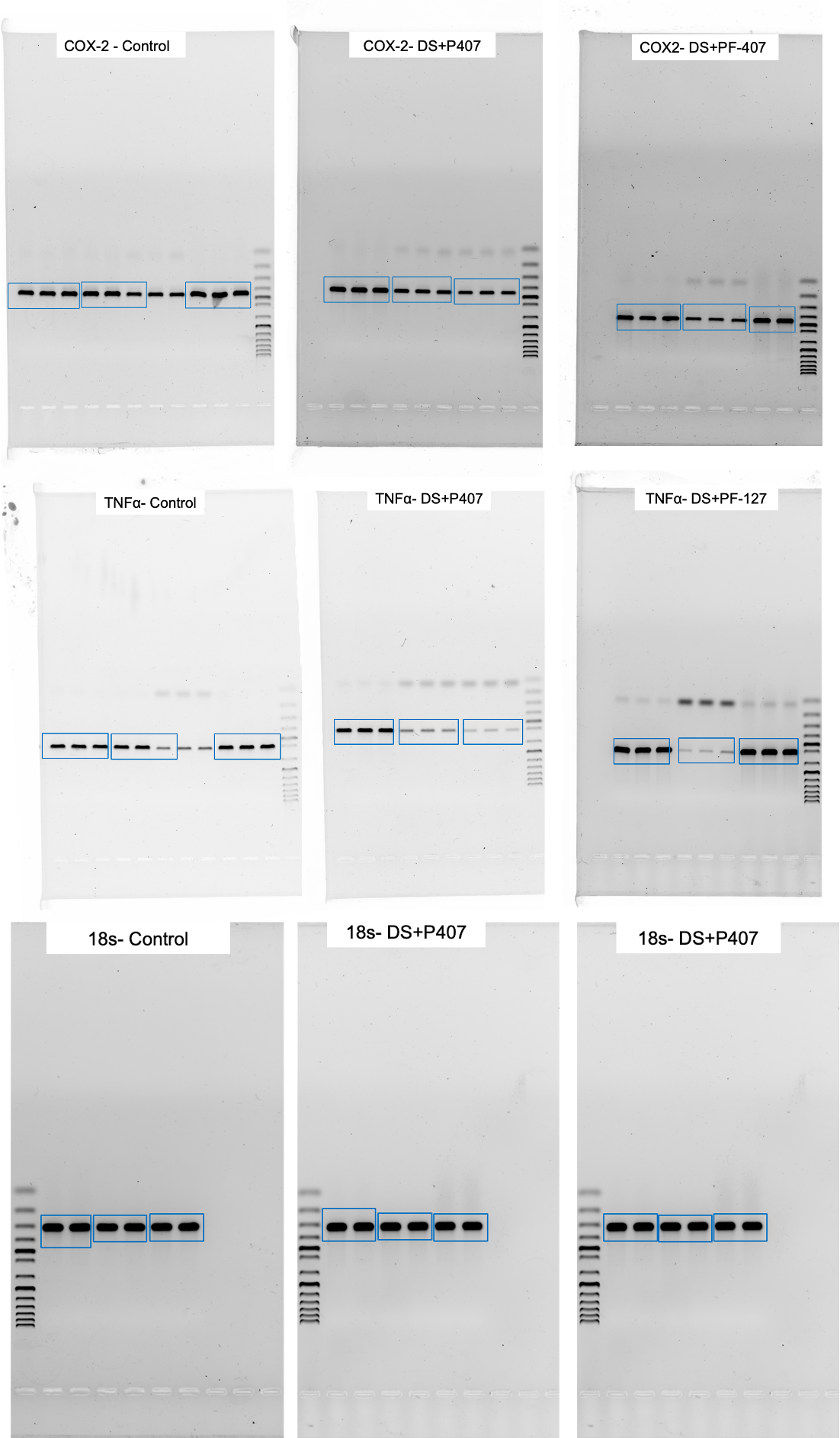


**Fig. S-5.** Full-length RT-PCR gel images of the respective genes.

Supplement: Supplementary file 2 — Supplementary Material 2 [file 12896_2024_856_MOESM2_ESM.docx]
